# Supplementary material for: Artificial Intelligence-Assisted Processing of Anterior Segment OCT Images in the Diagnosis of Vitreoretinal Lymphoma
Source: Diagnostics (Basel). 2023 Jul 23;13(14):2451. doi: 10.3390/diagnostics13142451 (PMC10378347; doi:10.3390/diagnostics13142451)
Supplement: Supplementary file 1 [file diagnostics-13-02451-s001.zip › diagnostics-2406181-supplementary.pdf]

Supplementary material

Features correlation study

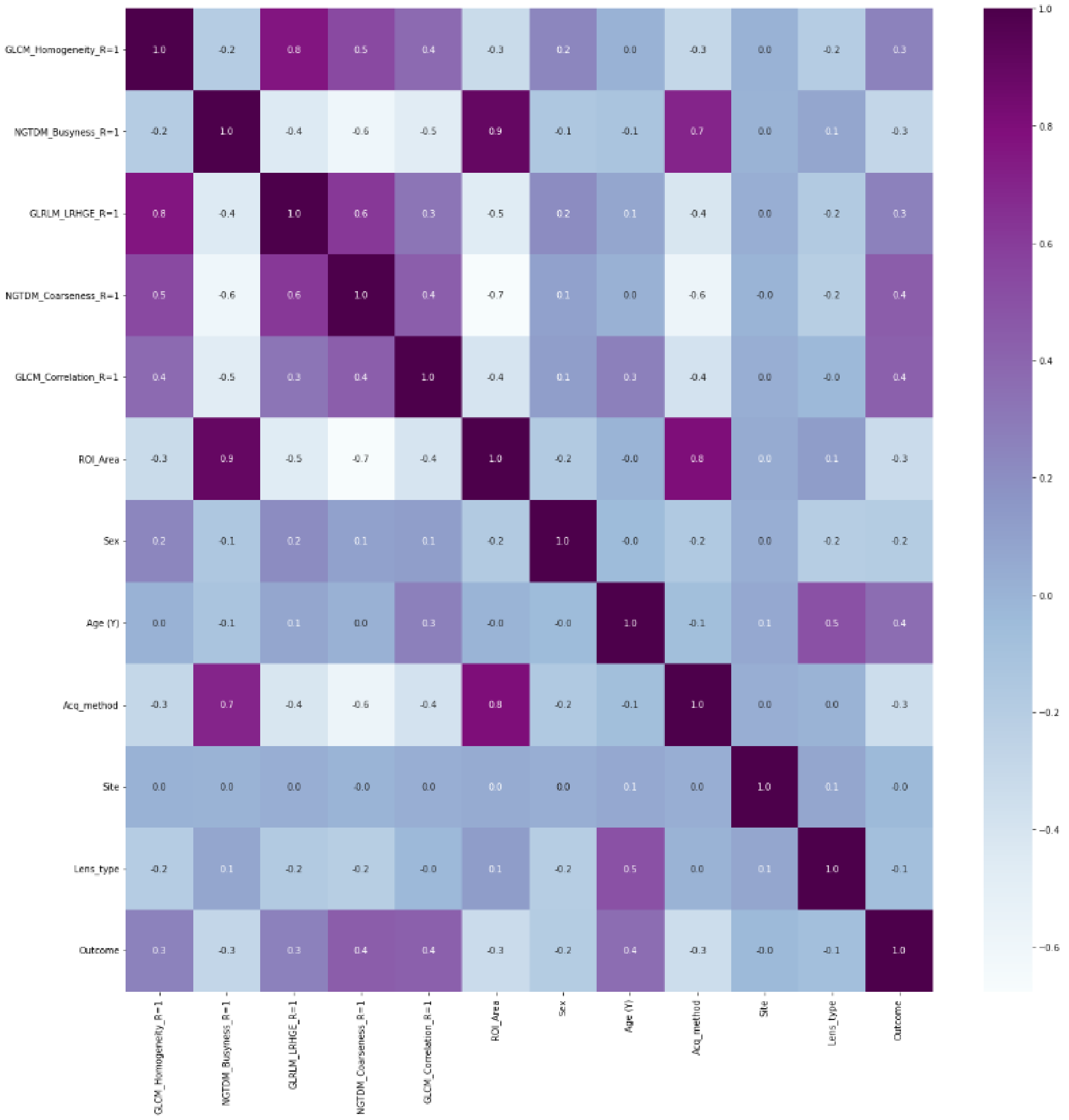

Figure S1: Pearson correlation coefficients (r) among the selected features and the other variables using 16 gray levels

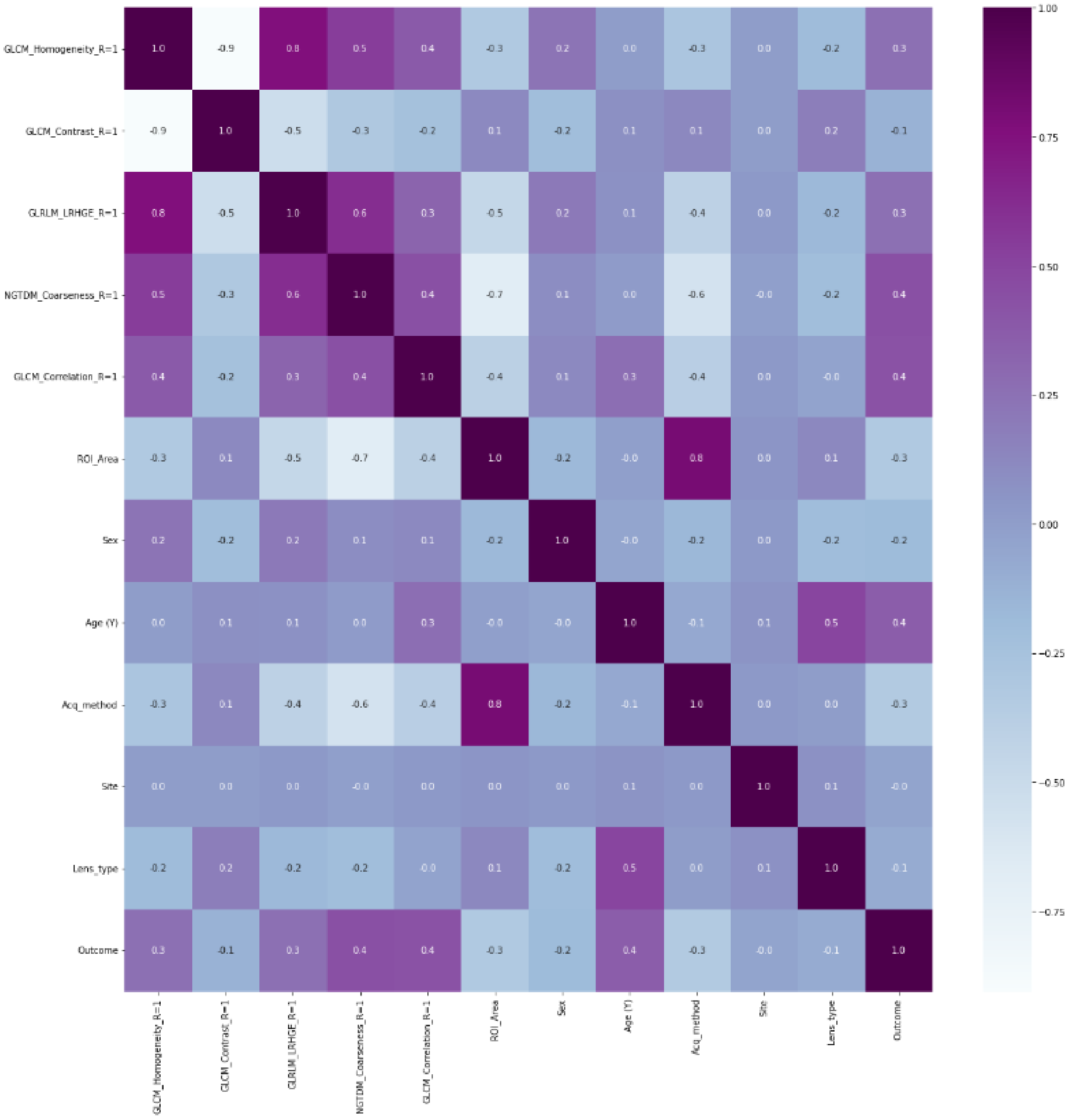

Figure S2: Pearson correlation coefficients (r) among the selected features and the other variables using 32 gray levels

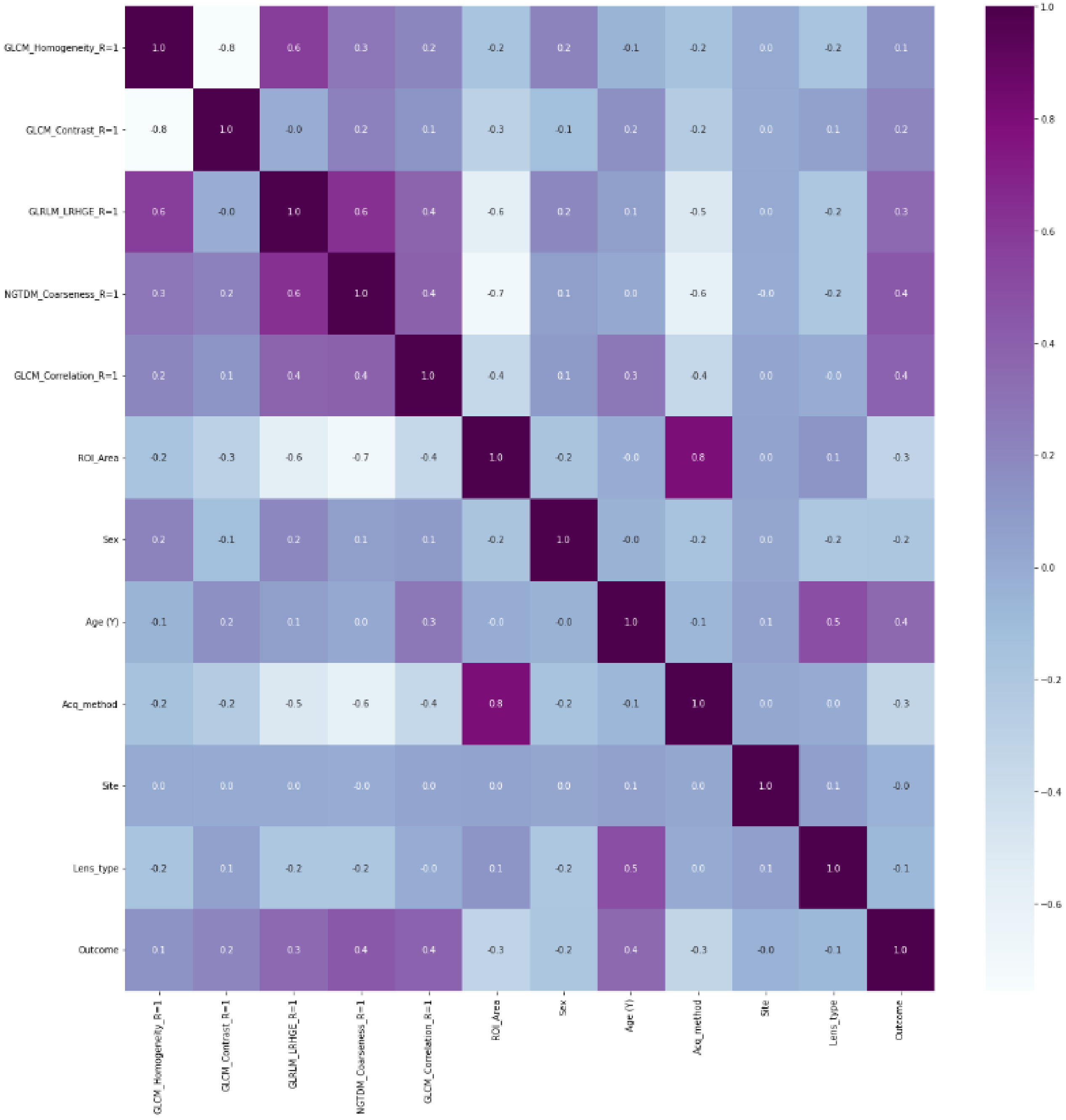

Figure S3: Pearson correlation coefficients (r) among the selected features and the other variables using 64 gray levels

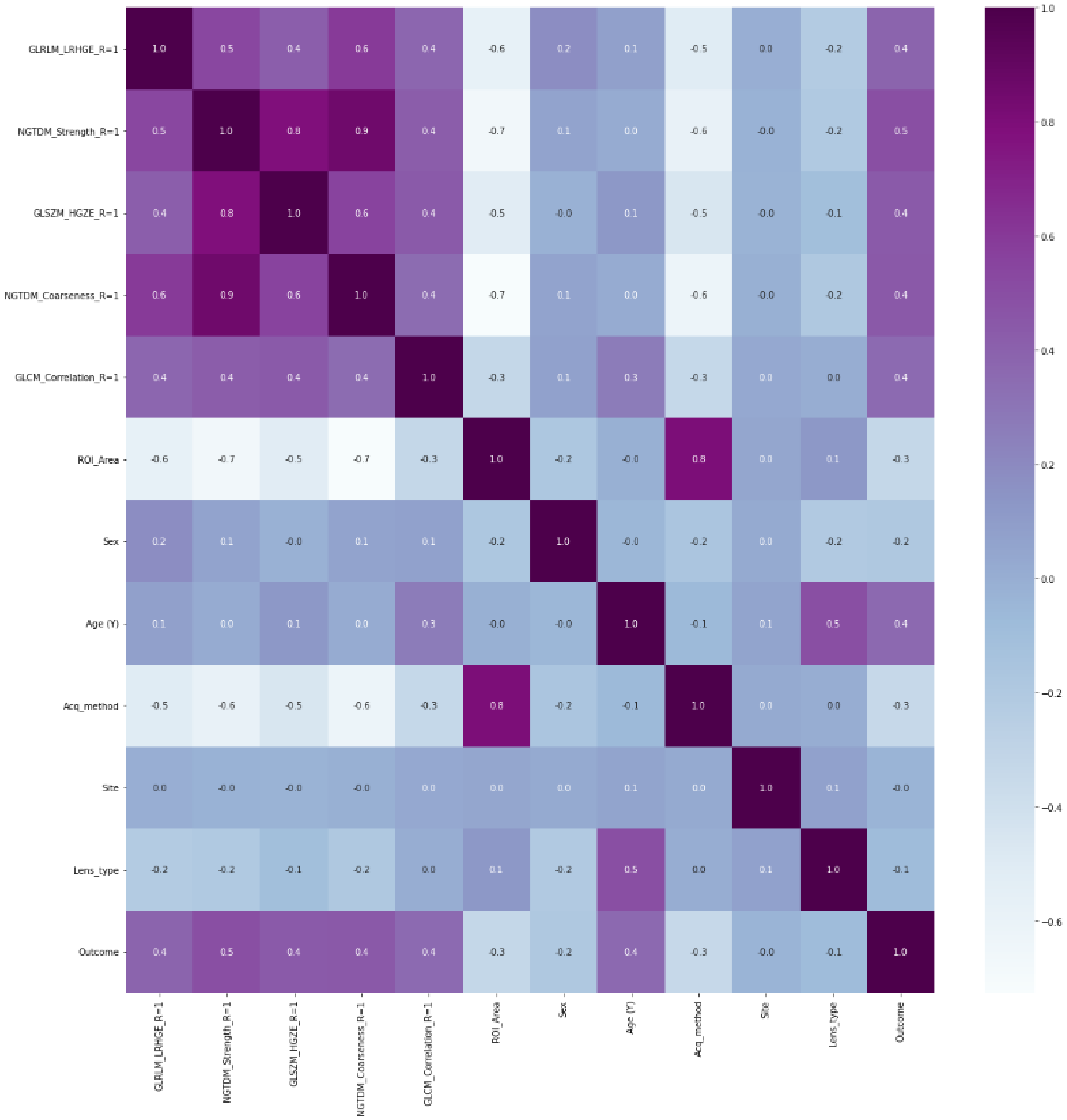

Figure S4: Pearson correlation coefficients (r) among the selected features and the other variables using 128 gray levels

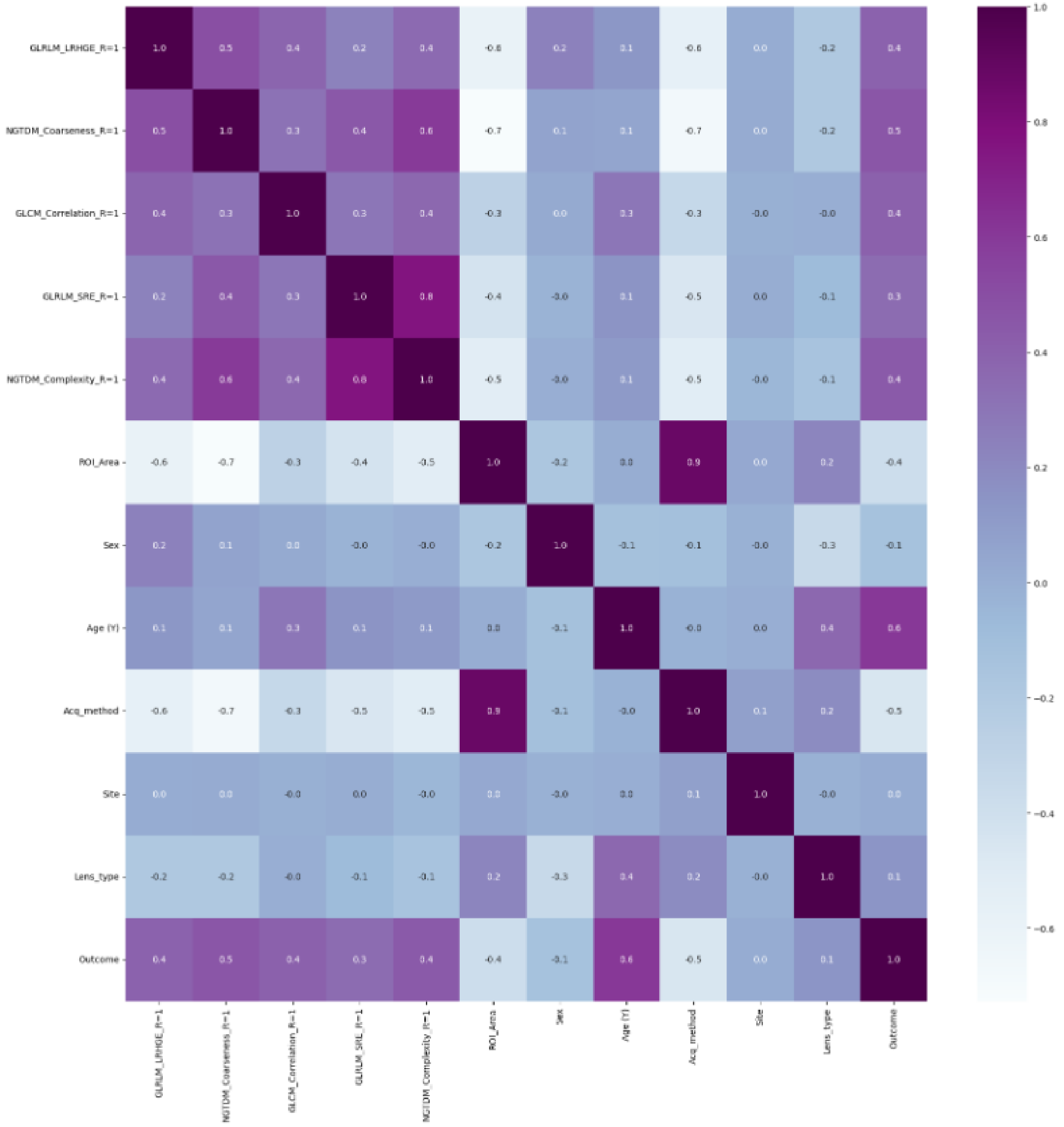

Figure S5: Pearson correlation coefficients (r) among the selected features and the other variables using 256 gray levels

Models results

Table S1: Model outcomes by patient in the training set. The number in brackets is the percentage of correctly detected images; if the percentage was less than 50%, there was a misclassification.

Patient code	Model	Model classification (% of correctly detected images)	True classification	Age (Y)
173	Dataset Ng = 16	VRL (100%)	VRL	51
	Dataset Ng = 32	VRL (97.2%)		
	Dataset Ng = 64	VRL (98.6%)		
	Dataset Ng = 128	VRL (98.6%)		
	Dataset Ng = 256	VRL (97.2%)		
186	Dataset Ng = 16	VRL (100%)	VRL	58
	Dataset Ng = 32	VRL (100%)		
	Dataset Ng = 64	VRL (100%)		
	Dataset Ng = 128	VRL (100%)		
	Dataset Ng = 256	VRL (100%)		
364	Dataset Ng = 16	VRL (84.4%)	VRL	82
	Dataset Ng = 32	VRL (78.1%)		
	Dataset Ng = 64	VRL (71.9%)		
	Dataset Ng = 128	VRL (81.3%)		
	Dataset Ng = 256	VRL (68.8%)		
393	Dataset Ng = 16	VRL (92.9%)	VRL	91
	Dataset Ng = 32	VRL (75%)		
	Dataset Ng = 64	VRL (80.4%)		
	Dataset Ng = 128	VRL (78.6%)		
	Dataset Ng = 256	VRL (80.4%)		
405	Dataset Ng = 16	Vitritis (98.2%)	Vitritis	38
	Dataset Ng = 32	Vitritis (97.3%)		
	Dataset Ng = 64	Vitritis (100%)		
	Dataset Ng = 128	Vitritis (99.1%)		
	Dataset Ng = 256	Vitritis (100%)		
433	Dataset Ng = 16	Vitritis (99.2%)	Vitritis	26
	Dataset Ng = 32	Vitritis (100%)		
	Dataset Ng = 64	Vitritis (100%)		
	Dataset Ng = 128	Vitritis (100%)		
	Dataset Ng = 256	Vitritis (99.2%)		
434	Dataset Ng = 16	Vitritis (97%)	Vitritis	57
	Dataset Ng = 32	Vitritis (100%)		
	Dataset Ng = 64	Vitritis (100%)		
	Dataset Ng = 128	Vitritis (99%)		

	Dataset Ng = 256	Vitritis (99%)		
435	Dataset Ng = 16	Vitritis (98%)	Vitritis	20
	Dataset Ng = 32	Vitritis (100%)		
	Dataset Ng = 64	Vitritis (100%)		
	Dataset Ng = 128	Vitritis (100%)		
	Dataset Ng = 256	Vitritis (100%)		
436	Dataset Ng = 16	Vitritis (47.6%)	VRL	76
	Dataset Ng = 32	Vitritis (39.2%)		
	Dataset Ng = 64	Vitritis (39.8%)		
	Dataset Ng = 128	Vitritis (39.2%)		
	Dataset Ng = 256	Vitritis (38.6%)		
439	Dataset Ng = 16	Vitritis (99.2%)	Vitritis	74
	Dataset Ng = 32	Vitritis (100%)		
	Dataset Ng = 64	Vitritis (100%)		
	Dataset Ng = 128	Vitritis (100%)		
	Dataset Ng = 256	Vitritis (100%)		
440	Dataset Ng = 16	Vitritis (95%)	Vitritis	50
	Dataset Ng = 32	Vitritis (98%)		
	Dataset Ng = 164	Vitritis (98%)		
	Dataset Ng = 128	Vitritis (97%)		
	Dataset Ng = 256	Vitritis (98%)		
442	Dataset Ng = 16	VRL (100%)	VRL	88
	Dataset Ng = 32	VRL (100%)		
	Dataset Ng = 64	VRL (96.8%)		
	Dataset Ng = 128	VRL (100%)		
	Dataset Ng = 256	VRL (98.4%)		
444	Dataset Ng = 16	Vitritis (100%)	Vitritis	39
	Dataset Ng = 32	Vitritis (100%)		
	Dataset Ng = 64	Vitritis (100%)		
	Dataset Ng = 128	Vitritis (99%)		
	Dataset Ng = 256	Vitritis (100%)		
447	Dataset Ng = 16	Vitritis (44.2%)	VRL	94

Dataset Ng = 32	Vitritis (20%)
Dataset Ng = 64	Vitritis (36.8%)
Dataset Ng = 128	Vitritis (37.9%)
Dataset Ng = 256	Vitritis (33.7%)

Table S2: Model outcomes by patient in the testing set. The number in brackets is the percentage of correctly detected images; if the percentage was less than 50%, there was a misclassification.

Patient code	Model	Model classification (% of image correctly detected)	True classification	Age (Y)
103	Dataset Ng = 16	VRL (100%)	VRL	55
	Dataset Ng = 32	VRL (100%)		
	Dataset Ng = 64	VRL (100%)		
	Dataset Ng = 128	VRL (100%)		
	Dataset Ng = 256	VRL(100%)		
363	Dataset Ng = 16	VRL (100%)	VRL	71
	Dataset Ng = 32	VRL (100%)		
	Dataset Ng = 64	VRL (100%)		
	Dataset Ng = 128	VRL (100%)		
	Dataset Ng = 256	VRL(100%)		
398	Dataset Ng = 16	VRL (100%)	VRL	58
	Dataset Ng = 32	VRL (100%)		
	Dataset Ng = 64	VRL (100%)		
	Dataset Ng = 128	VRL (100%)		
	Dataset Ng = 256	VRL(100%)		
410	Dataset Ng = 16	VRL (24.8%)	Vitritis	79
	Dataset Ng = 32	VRL (35.8%)		
	Dataset Ng = 64	VRL (28.8%)		
	Dataset Ng = 128	VRL (49.6%)		
	Dataset Ng = 256	Vitritis (54.2%)		
432	Dataset Ng = 16	Vitritis (25.7%)	VRL	73
	Dataset Ng = 32	Vitritis (31%)		
	Dataset Ng = 64	Vitritis (28.8%)		
	Dataset Ng = 128	Vitritis (34.1%)		
	Dataset Ng = 256	Vitritis (31.8%)		
437	Dataset Ng = 16	Vitritis (81.2%)	Vitritis	26
	Dataset Ng = 32	Vitritis (81.6%)		
	Dataset Ng = 64	Vitritis (82.5%)		
	Dataset Ng = 128	Vitritis (84.6%)		
	Dataset Ng = 256	Vitritis (82.8%)		
438	Dataset Ng = 16	Vitritis (89.5%)	Vitritis	46
	Dataset Ng = 32	Vitritis (90.5%)		
	Dataset Ng = 64	Vitritis (93.7%)		

	Dataset Ng = 128	Vitritis (92.7%)		
	Dataset Ng = 256	Vitritis (93.7%)		
445	Dataset Ng = 16	Vitritis (83.5%)	Vitritis	52
	Dataset Ng = 32	Vitritis (83.3%)		
	Dataset Ng = 64	Vitritis (83.4%)		
	Dataset Ng = 128	Vitritis (76.4%)		
	Dataset Ng = 256	Vitritis (80%)		
448	Dataset Ng = 16	Vitritis (83.5%)	Vitritis	65
	Dataset Ng = 32	Vitritis (76.5%)		
	Dataset Ng = 64	Vitritis (77%)		
	Dataset Ng = 128	Vitritis (76.4%)		
	Dataset Ng = 256	Vitritis (77%)		
446	Dataset Ng = 16	Vitritis (81%)	Vitritis	66
	Dataset Ng = 32	Vitritis (78.2%)		
	Dataset Ng = 64	Vitritis (88.2%)		
	Dataset Ng = 128	Vitritis (81.8%)		
	Dataset Ng = 256	Vitritis (85%)		
466	Dataset Ng = 16	Vitritis (72.3%)	Vitritis	62
	Dataset Ng = 32	Vitritis (76.7%)		
	Dataset Ng = 164	Vitritis (72.3%)		
	Dataset Ng = 128	Vitritis (76%)		
	Dataset Ng = 256	Vitritis (76%)		
468	Dataset Ng = 16	VRL (22.6%)	Vitritis	78
	Dataset Ng = 32	Vitritis (50%)		
	Dataset Ng = 64	VRL (45.2%)		
	Dataset Ng = 128	VRL (44.4%)		
	Dataset Ng = 256	VRL (40.3%)		
491	Dataset Ng = 16	Vitritis (75.3%)	Vitritis	75
	Dataset Ng = 32	Vitritis (79.3%)		
	Dataset Ng = 64	Vitritis (80.5%)		
	Dataset Ng = 128	Vitritis (80%)		
	Dataset Ng = 256	Vitritis (82.6%)		
493	Dataset Ng = 16	Vitritis (79.5%)	Vitritis	79
	Dataset Ng = 32	Vitritis (83.3%)		
	Dataset Ng = 64	Vitritis (88.3%)		
	Dataset Ng = 128	Vitritis (86.3%)		
	Dataset Ng = 256	Vitritis (91.2 %)		

Table S3: Model classification by patient and eye site. Y: Yes, i.e., correctly classified eye. N: No, i.e., misclassified eye

Patient code	Age (y)	Training or testing set	Eye site	Model Ng = 16		Model Ng = 32		Model Ng = 64		Model Ng = 128		Model Ng = 256	
				Eye correctly classify	Patient correctly classified	Eye correctly classify	Patient correctly classified	Eye correctly classify	Patient correctly classified	Eye correctly classify	Patient correctly classified	Eye correctly classify	Patient correctly classified
103	55	Testing	L	Y	Y	Y	Y	Y	Y	Y	Y	Y	Y
			R	Y		Y		Y		Y		Y	
173	51	Training	L	Y	Y	Y	Y	Y	Y	Y	Y	Y	Y
			R	Y		Y		Y		Y		Y	
186	58	Training	L	Y	Y	Y	Y	Y	Y	Y	Y	Y	Y
363	71	Testing	L	Y	Y	Y	Y	Y	Y	Y	Y	Y	Y
			R	Y		Y		Y		Y		Y	
364	82	Training	R	Y	Y	Y	Y	Y	Y	Y	Y	Y	Y
393	91	Training	L	Y	Y	Y	Y	Y	Y	Y	Y	Y	Y
			R	Y		Y		Y		Y		Y	
398	58	Testing	L	Y	Y	Y	Y	Y	Y	Y	Y	Y	Y
			R	Y		Y		Y		Y		Y	
405	38	Training	R	N	N	N	N	Y	Y	Y	Y	Y	Y
410	79	Testing	L	N	N	N	N	N	N	N	N	N	N
			R	N		Y		Y		Y		Y	
432	73	Testing	L	N	N	N	N	N	N	N	N	N	N
			R	N		Y		Y		N		N	
433	26	Training	L	Y	Y	Y	Y	Y	Y	Y	Y	Y	Y
			R	Y		Y		Y		Y		Y	
434	57	Training	L	Y	Y	Y	Y	Y	Y	Y	Y	Y	Y
			R	Y		Y		Y		Y		Y	
435	20	Training	L	Y	Y	Y	Y	Y	Y	Y	Y	Y	Y
			R			Y		Y	Y	Y		Y	
436	76	Training	L	N	N	N	N	Y	N	Y	N	N	N
			R	N		N		N		N		N	

437	26	Testing	L	Y	Y	Y	Y	Y	Y	Y	Y	Y	Y
			R	Y		Y		Y		Y		Y	
438	46	Testing	L	Y	Y	Y	Y	Y	Y	Y	Y	Y	Y
			R	Y		N		Y		Y		Y	
439	74	Training	L	Y	Y	Y	Y	y	Y	Y	Y	Y	Y
			R	Y		Y		Y		Y		Y	
440	50	Training	L	Y	Y	Y	Y	Y	Y	Y	Y	Y	Y
442	88	Training	L	N	N	Y	Y	Y	Y	Y	Y	Y	Y
			R	N		Y		Y		Y		Y	
444	39	Training	L	Y	Y	Y	Y	Y	Y	Y	Y	Y	Y
445	52	Testing	L	Y	Y	Y	Y	Y	Y	Y	Y	Y	Y
			R	Y		Y		Y		Y		Y	
446	66	Testing	L	Y	Y	Y	Y	Y	Y	Y	Y	Y	Y
447	94	Training	R	N	N	N	N	Y	Y	N	N	N	N
448	65	Testing	L	Y	Y	Y	Y	Y	Y	Y	Y	Y	Y
			R	Y		Y		Y		Y		Y	
466	62	Testing	R	Y	Y	Y	Y	Y	Y	Y	Y	Y	Y
468	78	Testing	L	N	N	Y	N	Y	N	Y	N	Y	N
			R	N		N		N		N		N	
491	75	Testing	L	Y	Y	Y	Y	Y	Y	Y	Y	Y	Y
			R	Y		Y		Y		Y		Y	
493	79	Testing	L	Y	Y	Y	Y	Y	Y	Y	Y	Y	Y
				% corrected eye detected	% corrected patients detected	% corrected eye detected	% corrected patients detected	% corrected eye detected	% corrected patients detected	% corrected eye detected	% corrected patients detected	% corrected eye detected	% corrected patients detected
				72%	75%	83%	79%	91%	89%	87%	82%	85%	82%

Models' ROC curves

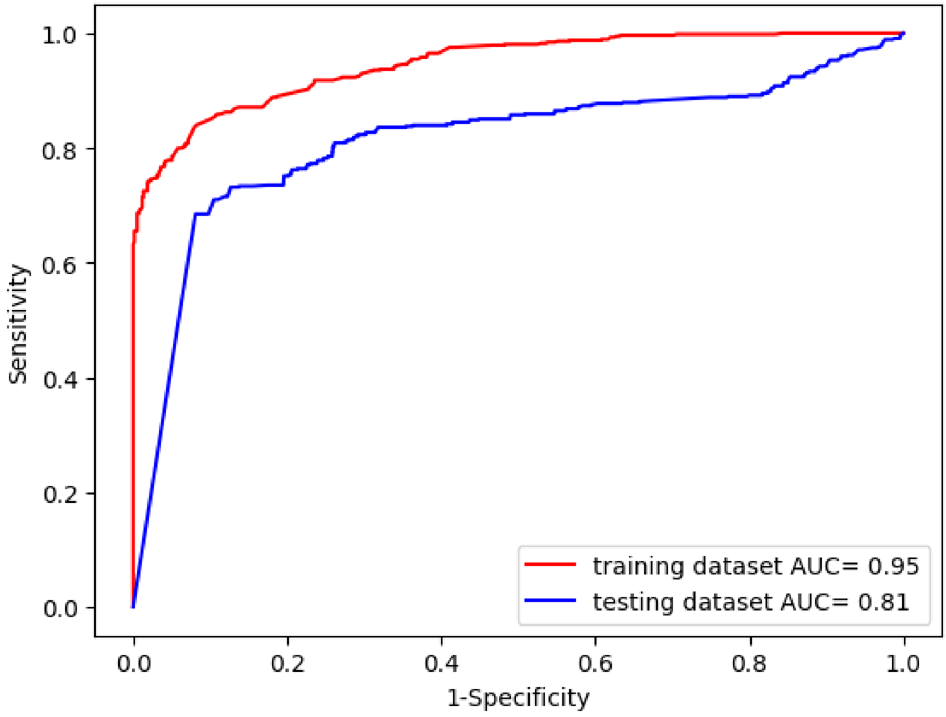

Figure S6: ROC curves obtained in training and testing sets with relative AUCs for model using "Dataset Ng = 16".

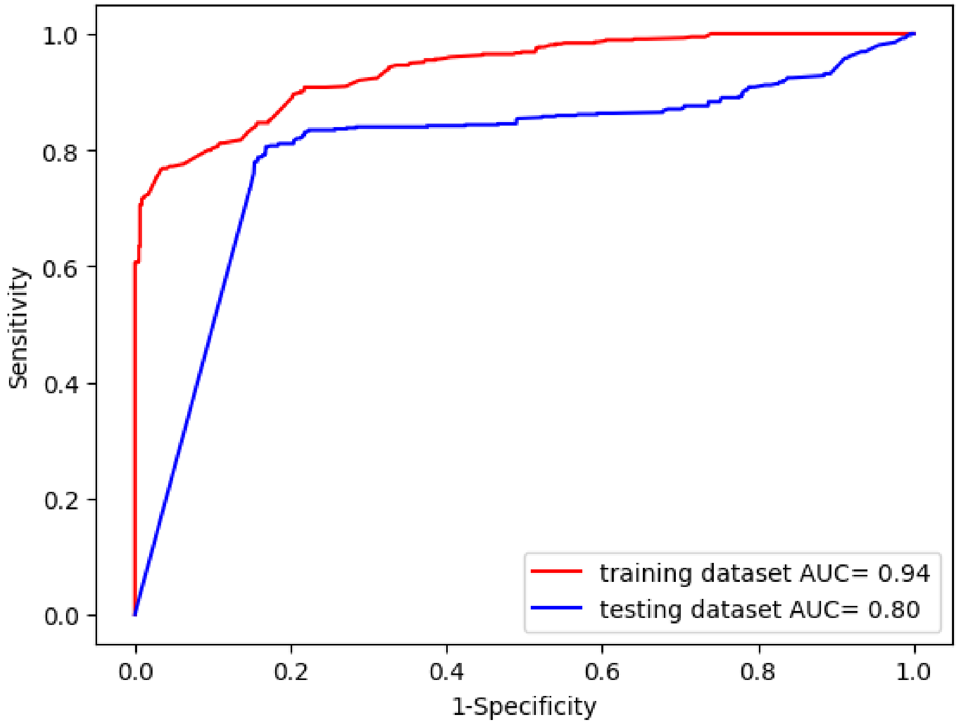

Figure S7: ROC curves obtained in training and testing sets with relative AUCs for model using "Dataset Ng = 32".

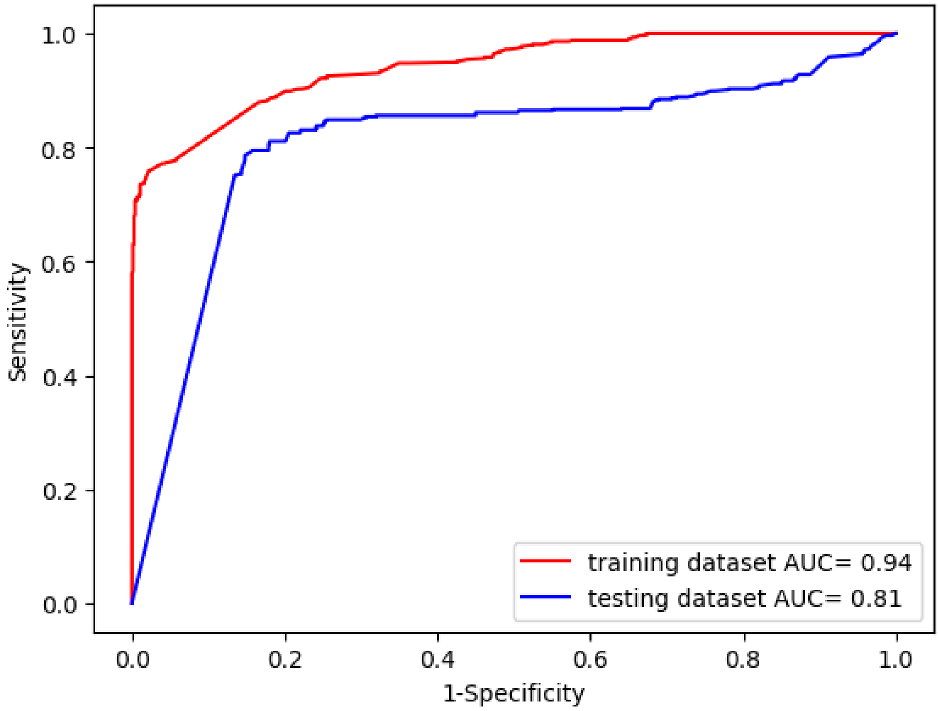

Figure S8: ROC curves obtained in training and testing sets with relative AUCs for model using “Dataset Ng = 64”.

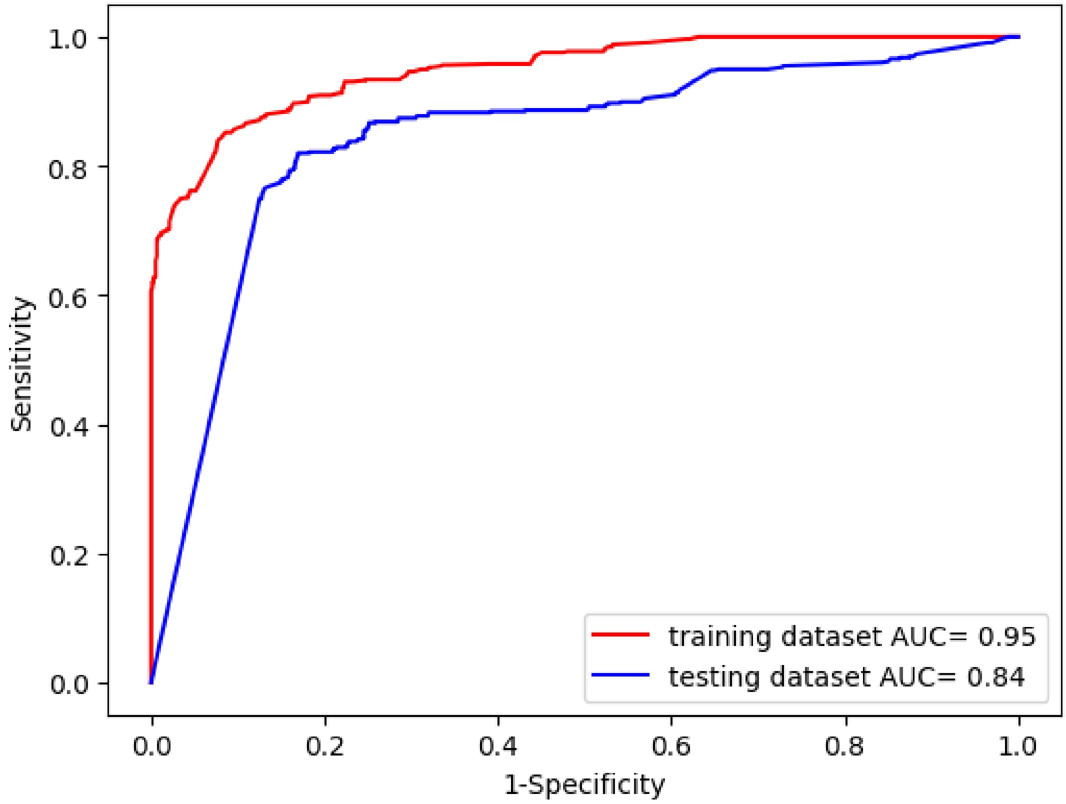

Figure S9: ROC curves obtained in training and testing sets with relative AUCs for model using “Dataset Ng = 128”.

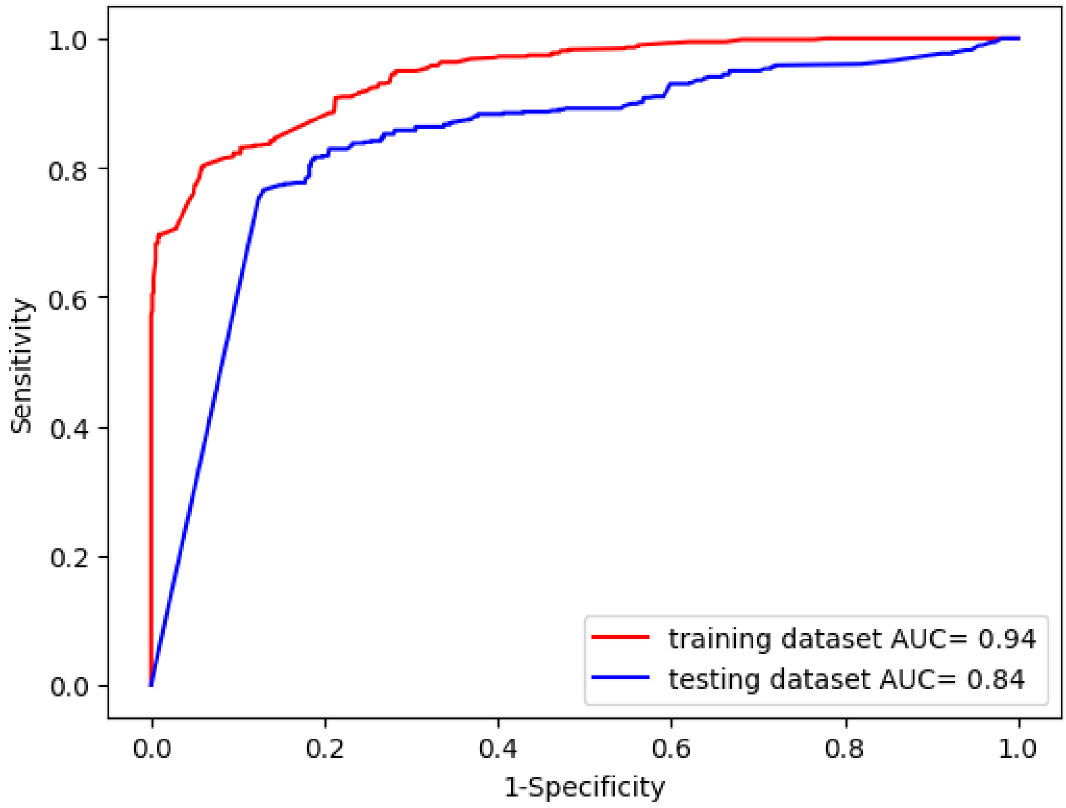

Figure S10: ROC curves obtained in training and testing sets with relative AUCs for model using “Dataset Ng = 256”.
